# Traumatic esophageal perforation in Puerto Rico Trauma Hospital: A case-series

**DOI:** 10.1016/j.amsu.2019.06.011

**Published:** 2019-06-28

**Authors:** Jan C. Vázquez-Rodríguez, Natalia M. Pelet del Toro, Omar García-Rodríguez, Ediel Ramos-Meléndez, Julio López-Maldonado, Felipe Rodríguez, Jorge Pelet Mejías, Pablo Rodríguez-Ortiz

**Affiliations:** a Ponce Health Sciences University, School of Medicine, Ponce, Puerto Rico; b University of Puerto Rico, School of Medicine, San Juan, Puerto Rico; c Division of Trauma and Critical Care Surgery, School of Medicine, University of Puerto Rico, San Juan, Puerto Rico

**Keywords:** Esophageal injury/perforation, Esophagus, Trauma, Blunt esophageal perforation, Gunshot esophageal perforation

## Abstract

**Background:**

Esophageal injuries are rare, life-threatening, events with an overall reported incidence of less than 3%. In rare cases, trauma due to blunt or penetrating injuries cause esophageal perforations, which account for less than 15% of all esophageal injuries.

**Materials and methods:**

A case-series study was conducted to describe the outcomes and management of all the traumatic esophageal injuries at the Puerto Rico Trauma Hospital (PRTH) from 2000 through 2017. These cases were evaluated in terms of etiology of perforation, mechanism of injury and esophageal level.

**Results:**

Sixteen patients were treated for esophageal injuries at the PRTH between 2000 and 2017. Of these patients, 15 (93.7%) were males with a median age of 24.5 years (16, 49). Regarding the etiology of the esophageal perforation, 2 (12.5%) patients suffered blunt esophageal trauma, and 14 (87.5%) patients had penetrating trauma to the esophagus. The most common mechanism of perforation was gunshot wound 10 (62.4%), followed by stab wound 4 (25.0%), and the least common were motor vehicle collision 1 (6.3%) and pedestrian injured by traffic 1 (6.3%). Regarding esophageal location, 9 (56.3%) patients presented cervical, 6 (37.5%) thoracic, and 1 (6.3%) abdominal injuries. Most patients 13 (81.3%) had a prompt diagnosis of traumatic esophageal perforation, while 3 (18.7%) patients had a delayed diagnosis. Only 2 (12.5%) deaths occurred among our 16 patients, including 1 (6.3%) in delayed diagnosed subjects.

**Conclusion:**

Esophageal perforation is a life-threatening condition and should be treated urgently. An early diagnosis and prompt surgical treatment completed in the first 24-h is fundamental for a good outcome.

## Highlights

• Sixteen patients were treated for esophageal injuries at the PRTH.
• Two patients suffered blunt esophageal trauma and 14 patients had penetrating trauma to the esophagus.
• Most patients had a prompt diagnosis of traumatic esophageal perforation, while 3 patients had a delayed diagnosis.
• Regarding esophageal location, of the 16 patients, 9 presented cervical, 6 thoracic, and 1 abdominal esophageal injury.
• Only 2 deaths occurred among our 16 patients, including 1 in delayed diagnosed subjects.

## 1 Introduction

Esophageal injuries are rare, life-threatening, events with an overall reported incidence of less than 3% [1]. The most common mechanism of esophageal injury is iatrogenic (70%), followed by spontaneous perforations [[1], [2], [3], [4], [5], [6]]. In rare cases, trauma due to blunt or penetrating injuries cause esophageal perforations, which account for less than 15% of all esophageal injuries [5,[7], [8], [9]]. The most common injury location is in the cervical esophagus (57%), followed by thoracic esophagus (26%) and abdominal esophagus (17%) [9]. Based on this criteria, the most frequent traumatic injury is a penetrating injury caused by firearms in the neck region [6,9].

An early diagnosis is crucial in the management of esophageal perforations, which is reported to have a 10–25% mortality rate. Therapy should be initiated within 24-h after the esophageal trauma; when treatment is delayed (>24-h), it would otherwise reflect a 40–66% mortality rate [5,7,[10], [11], [12], [13], [14], [15], [16]]. Esophageal injuries are usually managed with fluid resuscitation, mediastinal drainage, broad spectrum antibiotics and control of sepsis. Regardless of the injury location, a primary repair is preferred only for external injury and late presenting patients with esophageal trauma [[12], [13], [14], [15], [16], [17]].

Regarding diagnostic methods, no single approach has been described as useful for proper diagnosis of esophageal perforation since the methods barely describe any other clinical feature [9,12,17,[18], [19], [20], [21]]. In terms of the approach, patients who quickly progress to septic shock are treated surgically. Nevertheless, non-operable management, such as antibiotics, is administered to patients with no signs of infection or uncontained perforation [20,23].

There is limited evidence of this daunting surgical challenge, which contributes to its common delay in diagnosis and, consequently, its poor management strategies. A significant number of published research focuses on case reports which provide a small insight into the event [20,22]. This study aimed to define and evaluate the population of patients with traumatic esophageal perforations at the Puerto Rico Trauma Hospital (PRTH). According to Pascual-Marrero et al. (2017), Puerto Rico has an incidence of penetrating trauma of 27% compared to Europe which has reported an overall incidence of 9% [25]. PRTH is the only trauma hospital in Puerto Rico, serving a population of 3.1 millions. According to epidemiological studies, patients arrive with a higher injury severity score (ISS) than trauma centers in the United States (US) and take on average more than 4 h to arrive at the Trauma Bay [25]. This study will provide a better insight into the management and treatment of esophageal perforations.

The development of proper management procedures for these trauma patients is a significant public health issue due to its repercussions in patient disabilities and substantial increases in morbidity and mortality rates [24]. It has been well documented that the management and treatment of esophageal perforation are profoundly influenced by surgeon experience and judgment [20,[22], [23], [24]].

## 2 Methods

A case-series study was conducted to describe the outcomes and management of all the traumatic esophageal injuries at the PRTH from 2000 through 2017. The sample data was collected from medical records and the Trauma Registry, which is part of the National Trauma Registry System (NTRS) in the US. We included patients admitted to the hospital from January 2000 to December 2017 with the diagnosis of esophageal perforation based on ICD-9 and ICD-10 codes.

These cases were evaluated in terms of etiology of perforation (blunt or perforating), mechanism of injury (gunshot wound [GSW], stab wound [SW], pedestrian vs. automobile, or motor-vehicle collision [MVC]), and esophageal level (cervical, thoracic, or abdominal). Diagnostic management of esophageal perforation was based on esophagogram and computerized tomography (CT Scan). Additionally, the intraoperative findings and postoperative condition were included accordingly. Other information collected includes age, sex, admission to intensive care unit (ICU), length of stay (LOS) in the hospital, diagnostic methods, surgical procedures, postoperative complications, perforation size, delay in diagnosis, toxicology, ISS, Glasgow coma scale (GCS), and mortality.

Sample description was done using medians and maximum and minimum values for continuous variables and measures of absolute and relative frequencies (n and percentages) for categorical variables. Case series reported in line with PROCESS criteria [35]. The study protocol was approved by the Institutional Review Board of the Medical Sciences Campus of the University of Puerto Rico.

## 3 Results

Sixteen patients were treated for esophageal injuries at the PRTH between 2000 and 2017. Of these patients, 15 (93.7%) were males with a median age of 24.5 years (16, 49). Regarding the etiology of the esophageal perforation, 2 (12.5%) patients suffered blunt esophageal trauma, and 14 (87.5%) patients had penetrating trauma to the esophagus. The most common mechanism of perforation was GSW 10 (62.4%), followed by SW 4 (25.0%), and the least common were MVC 1 (6.3%) and pedestrian injured by traffic 1 (6.3%). Most patients had a prompt diagnosis of traumatic esophageal perforation 13 (81.3%), while 3 patients (18.7%) had a delayed diagnosis. This information is summarized in Table 1.

**Table 1:** Sociodemographic profile, drug use, and preoperative laboratory tests (N = 16).

Characteristic	n (%)
Age (years)	
Median (Min., Max.)	24.5 (16, 49)
Sex:	
Male	15 (93.7%)
Female	1 (6.3%)
Etiology of perforation:	
Blunt	2 (12.5%)
Penetrating	14 (87.5%)
Specific Mechanism:	
GSW	10 (62.4%)
SW	4 (25.0%)
Pedestrian vs. Auto	1 (6.3%)
MVC	1 (6.3%)
Ethanol:	
Yes	4 (25.0%)
No	12 (75.0%)
Marijuana:	
Yes	4 (25.0%)
No	12 (75.0%)
Cocaine:	
Yes	3 (18.7%)
No	13 (81.3%)
Benzodiazepine:	
Yes	4 (25.0%)
No	12 (75.0%)
Opiate:	
Yes	1 (6.3%)
No	15 (93.7%)
WBC (x10E3/uL)	
Median (Min., Max.)	20.1 (6.6, 31.5)
Hgb (g/dL)	
Median (Min., Max.)	13.2 (5.7, 15.2)
Neutrophils (%)	
Median (Min., Max.)	79.3 (54.0, 89.8)
Albumin (g/dL)	
Median (Min., Max.)	3.3 (2.2, 4.4)
Amylase (U/L)	
Median (Min., Max.)	77 (37, 913)
Lipase (U/L)	
Median (Min., Max.)	24 (6, 94)
pH	
Median (Min., Max.)	7.34 (7.21, 7.44)
Base Excess (mEq/L)	
Median (Min., Max.)	−3.2 (−9.0, 1.8)
Delay in Diagnosis (hours):	
>24	3 (18.7%)
≤24	13 (81.3%)

GSW: gunshot wound; SW: stab wound; MVC: motor vehicle collision; Hgb: hemoglobin; WBC: white blood cells.

Half of the patients arrived with an ISS ≥25 and 13 (81.3%) had a GCS > 8. The most common drugs among the patients were cocaine, benzodiazepine and ethanol, with a frequency of 25%. The toxicology report is summarized in Table 1. The median LOS and ICU days were 31.5 (11, 82) and 19 (2, 78) days, respectively. Trauma severity and outcomes are summarized in Table 3. Regarding esophageal location, of the 16 patients, 9 (56.3%) presented cervical, 6 (37.5%) thoracic, and 1 (6.3%) abdominal injuries (Table 2). Furthermore, 10(62.3%) patients underwent primary repair of the esophageal laceration. One primary repair was reinforced with a sternocleidomastoid muscle flap, and another underwent esophageal reconstruction with jejunal flap. The individual case report findings are shown in Table 2. The preoperative laboratory tests are described in Table 1.

**Table 2:** Cases of esophageal perforation admitted to Puerto Rico Trauma Hospital (N = 16).

	Injury Etiology	Esophageal Level	Preoperative Esophagogram	Surgical Procedure	Perforation Size	Postoperative Complications	Delay in Diagnosis	Mortality
1	GSW to the neck	Cervical	Yes	Primary Repair	1 cm Longitudinal + Transversal	Pancytopenia, Respiratory Failure, UTI	No	No
2	GSW to the neck	Abdominal	No	Primary Repair	Minimal	Pleural Effusion	No	No
3	GSW to the neck	Cervical	No	Primary Repair	3 cm Longitudinal	None	No	No
4	GSW to the neck	Cervical	No	Primary Repair	Minimal	Esophageal Leakage	No	No
5	GSW to the neck	Cervical	No	Primary Repair	1 cm Longitudinal	Pleural Effusion	No	No
6	GSW to neck and chest	Thoracic	No	Primary Repair	Minimal	Respiratory Failure, Septic Shock	No	Yes
7	GSW to the chest	Cervical	No	Primary Repair	2 cm Longitudinal	Pleural Effusion + ARDS	Yes	No
8	GSW to the chest	Thoracic	No	Esophageal Reconstruction with Jejunal Flap	2 cm Longitudinal	Pleural Effusion	Yes	No
9	GSW to the chest	Thoracic	No	Esophageal gastric anastomosis	Minimal	Pleural Effusion	No	No
10	GSW to the chest	Thoracic	Yes	Esophagectomy, cervical esophagostomy	3 cm Longitudinal + Transversal	Septic Shock, Respiratory Failure	No	No
11	SW to the chest	Thoracic	Yes	Esophageal Ligation + Esophagostomy infeeding jejunostomy	2 cm Transversal	Abscess	No	No
12	SW to the chest	Thoracic	No	Primary Repair	Minimal	Pleural Effusion	Yes	No
13	SW to the neck	Cervical	No	Esophagostomy infeeding jejunostomy	Minimal	Respiratory Failure	No	No
14	SW to the neck	Cervical	No	Primary Repair	Minimal	None	No	No
15	Blunt	Cervical	Yes	Primary Repair + Exploratory Laparotomy	2 cm Longitudinal + 1 cm Transversal	Pleural Effusion, Abscess, Respiratory Failure, Supraventricular Tachycardia, UTI	No	No
16	Blunt	Cervical	No	Primary Repair, reinforced with sternocleidomastoid patch	Minimal	Respiratory Failure, Atelectasis	No	Yes

GSW: gunshot wound; SW: stab wound.

**Table 3:** Trauma severity and outcomes (N = 16).

Characteristic	n (%)
ICU days	
Median (Min., Max.)	19 (2, 78)
LOS	
Median (Min., Max.)	31.5 (11, 82)
GCS	
>8	13 (81.3%)
≤8	3 (18.7%)
ISS	
≥25	8 (50.0%)
<25	8 (50.0%)
Mortality	
Yes	2 (12.5%)
No	14 (87.5%)

ICU: intensive care unit; LOS: length of stay; GCS: Glasgow coma scale; ISS: injury severity score.

Of the 3 patients with a delayed diagnosis, 1 (33.3%) had a complication of acute respiratory distress syndrome and pleural effusion. The other 2 (66.7%) patients only presented with pleural effusion. Only 2 (12.5%) deaths occurred among our 16 patients, including 1 (6.3%) in delayed diagnosed subjects. A total of 4 (25%) patients underwent exploratory thoracotomy and exploratory laparotomy.

## 4 Discussion

Esophageal perforation due to a traumatic surgical event has been reported as a life-threatening condition, and the adequate choice of treatment varies with the time of diagnosis, the extent of the perforation, etiology of perforation, judgment of the attending physician and any underlying conditions present.

As reported by literature, we found that the most common cause of traumatic esophageal perforation is due to a GSW, followed by SW and automobile accidents (car crash with injured motorcyclist and pedestrian injured by traffics) [[26], [27], [28], [29], [30]]. Concerning anatomic location, our findings are consistent with the scientific evidence, reporting cervical esophageal perforation as the most common type [18]. Cervical esophageal perforation has been described as less deleterious because the propagation of contamination is constrained in comparison with intrathoracic esophageal perforation [[27], [28], [29], [30]]. Cervical esophageal perforations have a lower mortality rate than thoracic and abdominal esophageal injuries. Our study reported 9 cervical esophageal perforations. Two of them did not experience any postoperative complications; and none of our cervical cases developed septic shock, which is consistent with literature. According to Sancheti (2016), the majority of cervical esophageal perforations are treated with primary repair of the esophageal lesion [32]. Of the 9 cervical esophageal perforations in our sample, 8 patients underwent primary repair of the esophageal lesion.

An increase in morbidity and mortality is evident in intrathoracic perforation. Since the mucosa and gastroesophageal content will drain into the mediastinal cavity, it will lead to infections [[27], [28], [29], [30]]. We reported 6 thoracic esophageal cases, of which only 1 developed septic shock and another had an esophageal abscess. Furthermore, 2 patients with thoracic esophageal perforation had a delayed in diagnosis, and developed a postoperative pleural effusion.

In order to minimize complications, an early diagnosis should be done, especially in thoracic esophageal perforation. Regarding abdominal esophageal perforation, our study reported 1 perforation in the lower esophagus with a postoperative complication of pleural effusion; in this case, the diagnosis was made within the 24-h after presentation to our hospital. Most of our patients had a lower esophageal injury that was diagnosed early and had primary repair via laparotomy. Imaging studies were coordinated after surgery to evaluate repair before oral feedings. Of the subset of patients who underwent thoracotomy, the mediastinum and pleura were opened and connected, and after the repair, they were drained with chest tubes. Even though T-tube repair is certainly an effective operative option, most of our patients were able to undergo esophagorrhaphy because of an early diagnosis and were buttressed when appropriate (for example with pedicled intercostal muscle flap). T-tube repair was considered but not required in our patients. One patient underwent a combined approach with thoracotomy and laparotomy and had feeding jejunostomy placed. Two other patients had feeding jejunostomy placed during initial ELAP. Patients had a nasogastric/orogastric tube placed intraoperatively. Our trauma patients represent a young population, which in most cases were not malnourished and could tolerate a few days NPO to evaluate repair with appropriate imaging. In the case of malnourished patients (preoperatively), we would be more aggressive and consider early jejunostomy.

Esophageal perforation treated within the first 24-h has a 10–25% mortality rate. However, a late diagnosis increases mortality to 40–66% [28]. Following the standard surgical management of esophageal perforations, all of our patients but 3 were treated within the first 24-h after presentation [2,7,12]. Only 2 mortalities were reported in the cases evaluated in this study. Both patients developed septic shock, and this might be due to their delayed presentation to our hospital from the periphery hospitals.

Due to its low incidence, physician's lack of clinical expertise has been a concern in the proper diagnosis of esophageal perforation. In the cases evaluated, half of the patients arrived with an ISS ≥25 and 13 had a GCS >8. The GCS reported is from the initial evaluation and before administration of any drugs. The GCS was found higher than expected, this could be explained by the injury pattern (penetrating injuries to body areas other than the head of the patient). The high ISS can delay the diagnosis of esophageal injury due to several overlying traumas present in the patients. With a score greater than 8 on the GCS, the patient does not show precise signs of how ill he/she is initially, making it more difficult to properly diagnose an esophageal perforation.

Many authors have encouraged numerous treatment protocols which include conservative therapy and proper surgical management depending on the severity of the case. The diagnostic method is limited to images and refined attention to nonspecific symptoms [29]. A contrast-enhanced esophagogram is used to diagnose esophageal perforation. It is more sensitive within 24-h post esophageal trauma. It has less than 10% false-negative results. The false negative results can be due to edema and inflammatory reaction secondary to esophageal injury. According to Andrade-Alegre (2005), the use of esophagogram for a proper diagnosis should be forward-looking. However, there are several concerns about possible inflammatory reactions in the mediastinum when barium contrast is used; therefore, it should be limited [27]. The most commonly used contrast is gastrografin due to minimal inflammatory reactions such as mediastinitis. However, gastrografin swallow test has a sensitivity of 33% [28]. In our cases, only 4 patients had a preoperative esophagogram as a diagnostic method. Other diagnostic methods, such as endoscopy, have been controversial and should only be considered when images are negative and a high index of suspicion persists [29]. An endoscopy can cause another iatrogenic perforation. Although endoscopy is certainly an alternative on stable patients, it only has a positive predictive value of approximately 33% [36]. In our institution, CT Scan and esophagogram are more accessible to the patient than endoscopy.

This was observed because patients either had clear indications for surgery or CT scan with IV/PO contrast showed findings that were suspicious for perforation or other traumatic injuries, requiring emergency surgery and further investigation for possible esophageal injury in the operating room. In stable patients, signs in chest x-ray such as pleural effusions, subcutaneous emphysema and pneumo-mediastinum could facilitate a diagnosis of esophageal perforation [37].

Our findings are consistent with the recent literature, reporting a radical decrease in mortality for patients with esophageal perforation [[28], [29], [30], [31]]. This reduction is due to modern surgical techniques, new technology, advanced diagnostic methods, as well as the better insight of surgeons [28].

In terms of hospital and ICU LOS, we reported a median of 31 and 19 days, respectively. The expenses in diagnosis and treatments related to esophageal injury are very high. Making an early and accurate diagnosis can significantly reduce these expenses. This can be achieved by having a properly trained team able to diagnose an esophageal perforation in a timely manner. This will diminish complications and, subsequently, LOS in the hospital [33].

This study has several limitations, particularly our constrained sample of 16 patients from 2000 to 2017. The data was strictly collected from the Trauma Registry, operation reports and postoperative notes. An important limitation in the NTRS arises from the lack of documentation and disparity in recording essential information. Moreover, the lack of prehospital information, such as vitals at the trauma scene, represents another limitation for the investigator [34].

## 5 Conclusion

Esophageal perforation is a life-threatening condition and should be treated urgently. Our study demonstrated that early diagnosis and prompt surgical treatment completed in the first 24-h is fundamental to achieve a good outcome after esophageal perforation. In our patients, CTA with oral contrast was valuable in diagnosing esophageal perforation promptly, in addition to the other injuries associated with the patient's trauma. This proved to be very helpful in our trauma patients, half of which had an ISS ≥25. Most of the patients in which a delay in diagnosis was seen, were patients who took a considerable time to arrive at the PRTH. This should urge a look into the Puerto Rican prehospital management logistics to identify what can be done to expedite the time of arrival of the trauma patients to our hospital, which is the only Trauma Hospital in Puerto Rico. Further investigation should endeavor at how other Trauma Centers are managing these traumatic esophageal perforation patients and attempt to create a multihospital algorithm for managing this condition. With a more extensive study population that is more heterogeneous, standard guidelines could be developed that could be used around the US mainland and all its territories. Further research also can be done on the effect of ISS or patient comorbidities in the outcomes of these patients.

## Provenance and peer review

Not commissioned, externally peer reviewed.

## Consent

Not applicable.

## Ethical approval

This case series was approved by the Institutional Review Board of the University of Puerto Rico Medical Sciences Campus.

Protocol Number: B0030814.

## Conflicts of interest

No conflicts of interest to disclose.

## Sources of funding

No any sources of funding.

## Author contribution

Pablo Rodríguez, MD – Project administration; supervisor.

Jan C. Vázquez – writing – review and editing/data curation.

Natalia Pelet – writing – review and editing/data curation.

Omar Garcia - writing, data curation and formal analysis.

Ediel Ramos – writing, data curation and formal analysis.

Felipe Rodríguez, MD – data curation.

Julio López, MD – writing – review and editing/data curation.

Jorge Pelet, MD – writing – review and editing.

Lourdes Guerrios, MD - supervisor.

## Registration of research studies

UIN: researchregistry4752.

## Guarantor

Jan C. Vázquez-Rodríguez – accepts fully responsibility for this case-series. Contact via e-mail: vazquezjanc@gmail.com.
